# Electrically Controlled Membranes Exploiting Cassie-Wenzel Wetting Transitions

**DOI:** 10.1038/srep03028

**Published:** 2013-10-23

**Authors:** Edward Bormashenko, Roman Pogreb, Sagi Balter, Doron Aurbach

**Affiliations:** 1 Ariel University, Physics Faculty, 40700, P.O.B. 3, Ariel, Israel; 2 Ariel University, Department of Chemical & Biotechnology Engineering, P.O.B. 3, 40700, Ariel, Israel; 3 Department of Chemistry, Bar-Ilan University, 52900 Ramat-Gan, Israel

## Abstract

We report electrically controlled membranes which become permeable when an electrical field is exerted on a droplet deposited on the membrane. Micro-porous polycarbonate membranes are obtained with the breath-figures assembly technique, using micro-scaled stainless steel gauzes as supports. The membranes demonstrate pronounced Cassie-Baxter wetting. Air cushions trapped by the droplet prevent water penetration through the membrane. We demonstrate two possibilities for controlling the permeability of the membrane, namely contact and non-contact scenarios. When an electrical field is exerted on a droplet deposited on the membrane, the triple-line is de-pinned and the wetting transition occurs in the non-contact scheme. Thus, the membrane becomes permeable. The contact scheme of the permeability control is based on the electrowetting phenomenon.

Electrically controlled membranes attracted the attention of investigators in light of their applications for microfluidics, slow-release drug delivery, transport of proteins and DNA^1^,^2^,^3^,^4^,^5^,^6^. We propose a novel approach to the elaboration of electrically controlled membranes, namely exploiting Cassie-Wenzel transitions induced by imposing an electric field on a droplet deposited on a porous permeable polymer substrate. It is well-known that exerting an electric field may modify the wetting regime of a solid/liquid pair^7^,^8^,^9^,^10^,^11^. This takes place in electrowetting experiments in which the surface tension at the solid/liquid interface is influenced by an electric field^8^,^9^,^10^,^11^. In the classical electrowetting scheme a droplet is located between two electrodes, one of which touches the droplet (see Fig. 1a)^7^. In the electrowetting-on-dielectric (EWOD) scheme of electrowetting a droplet contacts with a dielectric layer coating the electrode^7^,^12^,^13^,^14^,^15^. The EWOD scheme allows significant decreases in the voltage necessary for the control of wettability. In our paper we demonstrate the possibility of exploiting the EWOD technique for controlling the permeability of polymer membranes assembled on micrometrically scaled metallic gauzes serving as electrodes.

We also propose a non-contact scheme of controlling the permeability of membranes in which electrodes do not touch a droplet, as shown in Fig. 1b. A droplet deposited on the micro-porous permeable surface is deformed by an external electric field^16^,^17^,^18^, and its triple (three-phase) line is detached from the substrate. Thus, the Cassie-Wenzel transition occurs^19^,^20^,^21^,^22^ and the liquid penetrates the membrane.

## Results

### Manufacturing membranes

The microscopically scaled membranes were prepared with the “breath-figures self-assembly” technique using stainless steel micro-porous gauzes as supports (as described in detail in the Methods section and Ref. 23). The SEM image of the gauze is presented in Fig. 2. The use of stainless steel micro-porous gauzes provided the water permeability of the membranes. Since the process of “breath-figures self-assembly” was reported by Widawski, Rawiso and Francois^24^, the theoretical and experimental activities in this field have been extended over the past decade^25^,^26^,^27^,^28^,^29^. Despite the fact that the physical mechanism of the patterning phenomenon is not clear to a full extent, the water-assisted self-assembly technique has already been successfully applied for manufacturing strictly ordered, closely packed micro- and nanoscale porous 2D structures^26^,^27^. Generally, the “breath-figures self-assembly” is related to the condensation of micro-scaled water droplets on the cooled surface of an evaporated polymer solution^25^,^28^. The droplets then sink into the solution, eventually forming the honeycomb pattern^25^,^26^,^27^,^28^. It was already demonstrated that polycarbonate (PC), which is an amorphous thermoplastic polymer, possessing high mechanical, electric and thermal properties (a glass transition temperature of about 147°C), may be effectively used for breath-figures self-assembly^29^,^30^.

PC membranes self-assembled with micro-porous stainless steel supports are depicted in Fig. 3. The average diameter of pores was about 5 μm.

20 μl distilled water droplets were deposited on the membranes. The membranes demonstrated pronounced Cassie-Baxter wetting^22^,^31^,^32^,^33^,^34^. The apparent contact angle was established as 125 ± 1°, whereas the static (or “as placed”, see Ref. 35) contact angle for the same solution deposited on atomically flat PC was 76 ± 1°. The Cassie-Baxter wetting regime means that a droplet is supported partially by polymer (PC) and partially by air^8^,^22^,^31^,^32^,^33^,^34^. This makes the membrane impermeable to water.

### Control of the permeability of membranes by electrowetting

**Figure 1:**
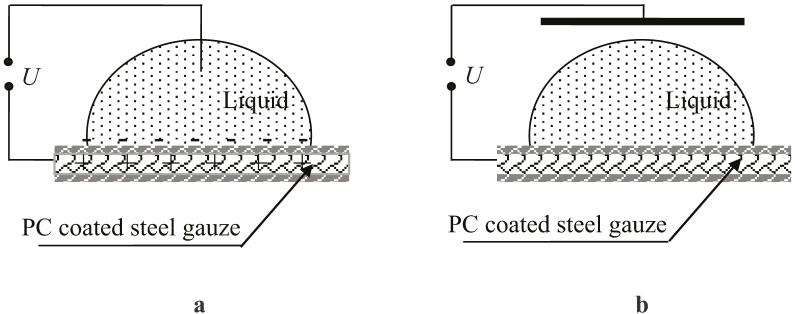
(a) Traditional scheme of an electro-wetting experiment. *U* is the voltage. The contact angle is changed due to formation of the double Helmholtz layer at the solid/liquid interface. The solid substrate in the PC coated gauze. (b) Non-contact scheme: the droplet deposited on the membrane is exposed to a uniform electric field.

We demonstrate the possibility to control the permeability of the membranes by exploiting the electrowetting effect^7^. When an aqueous electrolyte contacts a solid surface, a double electrical layer is formed^7^,^8^,^9^,^10^,^11^. The double layer works as a capacitor; thus the effective energy of the solid/liquid interface may be written as: 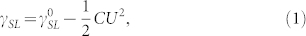
where 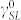 represents the solid/liquid interface tension at zero voltage, *C* is the capacitance per unit area of the substrate, and *U* is the voltage, applied between electrodes as shown in Fig. 1a. Substitution of Exp. (1) into the Young Formula: 
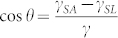
, yields for the electrowetting of a flat surface a contact angle *θ_el_*: 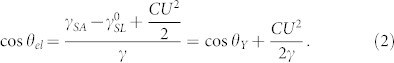
Generalization of Exp. (2) for rough and chemically heterogeneous surfaces (and this is the case in our study) was reported in Ref. 11. When a droplet is deposited on PC-coated gauze as shown in Fig. 1a, we have a modification of the EWOD scheme with the gauze serving as an electrode. We established that voltages as high as 250 V are necessary for noticeable change of the apparent contact angle inherent for wetting transitions^19^,^20^,^21^,^22^,^36^,^37^,^38^,^39^,^40^,^41^. This value of voltage corresponding to the onset of wetting transition coincides well with the electrowetting voltage necessary for the Cassie-Wenzel transition reported recently in Ref. 42. The noticeable penetration of water through the membrane was observed at voltages as high as 1 kV. Such high values of the voltage make the use of membranes problematic.

In order to decrease voltage, we performed hydrophilization of PC membranes with the cold air radiofrequency plasma, as described in detail in the Methods Section. The plasma treatment creates a complex mixture of surface functionalities which influence physical and chemical properties of the surface. In particular, it results in a dramatic change of wetting behavior, resulting in the hydrophilization of the surface^43^,^44^,^45^,^46^,^47^. The initial apparent contact angle of plasma treated membranes was of 70–80°. It should be emphasized that despite hydrophilization they remained impermeable for water.

After the treatment of membranes with the cold air radiofrequency plasma we succeeded in obtaining water penetration by applying a relatively low voltage of dozens of volts, as shown in Fig. 4. It could be seen from Fig. 5 that a sharp decrease in the contact angle was accompanied by an increase in the contact line radius occurring at 12 V. It is noteworthy that the decrease of the apparent contact angle, evidencing the wetting transition, was attended by the penetration of 5 μl of water through the membrane. Deformation of the droplet was accompanied by detachment (de-pinning) of the triple (three-phase) line of the droplet. The displacement of the triple line established with goniometer is illustrated in Fig. 5. The precise microscopic picture of the water penetration remained unknown, but it is reasonable to suggest that de-pinning of the triple line led to the wetting transition^19^,^20^,^21^,^22^,^36^,^37^,^38^,^39^,^40^,^41^. We established that the maximal displacement of the triple line Δ*R*_max_ equaled approximately 0.1*R*_0_ ~ 100 μm, hence the de-pinned triple line slipped over and filled a dozen lines of pores, as estimated very roughly.

### Non-contact electrical control of the permeability of membranes

A uniform electric field of 0–10 kV/cm was applied to the droplet, as shown in Fig. 1b. The electric field sufficient for deformation of the droplet was 2.5 kV/cm as shown in Fig. 6. Deformation of droplets by an electric field results from the complex interplay of electrical, surface tension and gravity-related effects^48^. The free energy *G* of the droplet exposed to the electric field could be estimated as: 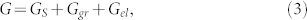
where *G_S_*, *G_gr_* and *G_el_* are surface, gravitational and electrical field-related contributions to the free energy respectively, *G_S_* ≅ *γS* ≅ *γ*4*πR*^2^, *G_el_* ≅ *ε*_0_*εE*^2^*V*/2, *G_gr_* = *ρVgh* ≅ *ρVgR*/2, where *R*, *S*, *V* and *h* are the characteristic dimension, surface, volume and the height of the center mass of the droplet respectively; *γ*, *ρ* and *ε* are surface tension, density and dielectric constant of a liquid respectively^48^. The dimensionless constants *ξ* and *χ* could be introduced as: 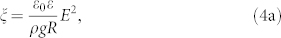


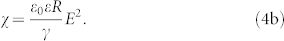
The dimensionless constant *ξ* describes the interrelation of gravity and electrical field-induced effects, and the constant *χ* describes the interrelation of surface tension-induced and electrical effects. Substituting *R* = 1 mm, *ρ* = 10^3^ kg/m^3^, *γ* = 70·10^−3^ J/m^2^, *ε* = 80 we obtain that when *E* ~ 1 kV/cm, *ξ* ~ 1, *χ* ~ 0.1. It means that in our case the electrical energy is mainly spent as the counteraction to gravity.

The deformation of a droplet was accompanied by the de-pinning of the triple line and water penetration through the membrane, as shown in Fig. 7. However, the displacement of the triple line was much less pronounced when compared to the electrowetting experiment. The maximal displacement of the triple line Δ*R*_max_ was equal to approximately 0.02*R*_0_ ~ 20 μm, hence in this case the de-pinned triple line slipped over and filled only a couple of the lines of pores (recall that the average size of the pore was of 5 μm).

## Discussion

The electrically controlled permeability of the membranes demonstrated in our study is based on the effect of the Cassie-Wenzel wetting transitions^19^,^20^,^21^,^22^,^36^,^37^,^38^,^39^,^40^,^41^,^42^. In our investigation wetting transitions were stimulated by exerting electric field on the liquid/porous solid system. One of the most debatable problems in the field of wetting is the problem of the "dimension" of the transitions, or, in other words: whether all pores underneath the droplet should be filled by liquid (the "2D scenario"), or perhaps only the pores adjacent to the three-phase (triple) line are filled under external stimuli such as pressure, vibrations or impact (the "1D scenario")^34^,^20^,^37^,^38^,^39^,^40^,^41^,^42^. Our investigation does not supply an unambiguous answer to this question, but it demonstrates explicitly that the wetting transition is accompanied by a micro-metrically scaled displacement of the triple line depicted in Figs. 5, 7. It is plausible to suggest that the Cassie-Wenzel wetting transitions are mainly governed by wetting events occurring in the nearest vicinity of the triple line, as demonstrated in a series of recent investigations^34^,^37^,^38^,^39^,^40^,^41^,^42^.

We conclude that electrically driven membranes obtained with breath-figures self-assembly allow a low voltage (~10 V) control of water penetration under conditions of electrowetting. The non-contact control of water penetration through membranes is also reported, however, it needs much higher voltage for the electrical deformation of a droplet. The permeability of membranes results from the Cassie-Wenzel wetting transitions, accompanied by the displacement of the triple line registered in both contact and non-contact schemes; however, the displacement of the triple line is more pronounced under conditions of the electrowetting experiment.

## Methods

**Figure 2:**
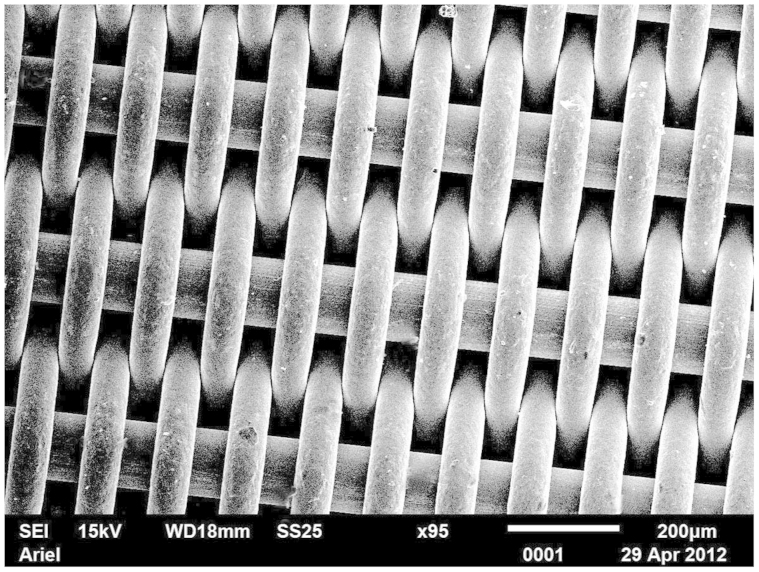
SEM image of the stainless steel wire gauzes used as supports for breath-figures self-assembly. The scale bar is 200 μm.

**Figure 3:**
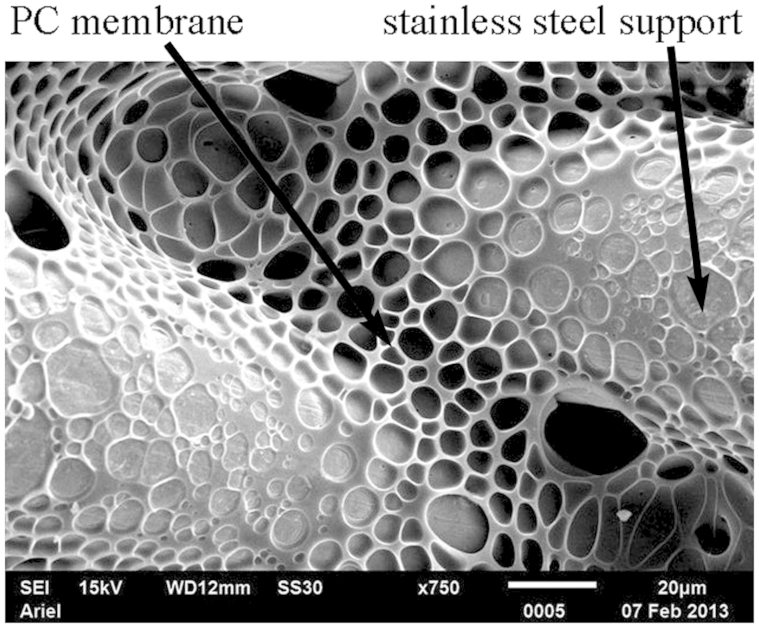
SEM image of the stainless steel gauzes coated with porous polycarbonate film with the breath-figures self-assembly. The scale bar is 20 μm.

**Figure 4:**
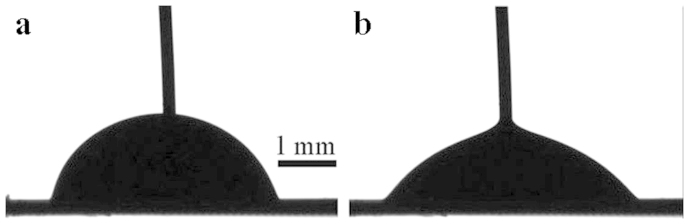
Opening the membrane by electrowetting. A 20 μl droplet deposited on the plasma treated PC-coated metal gauze: (a) *U* = 0 V; (b) *U* = 30 V.

**Figure 5:**
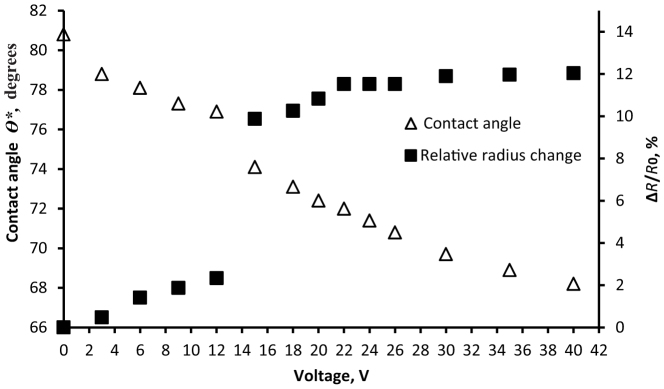
The apparent contact angle θ* and the dimensionless relative radius Δ*R*/*R*_0_ vs. applied voltage *U* in the electrowetting experiment.

**Figure 6:**
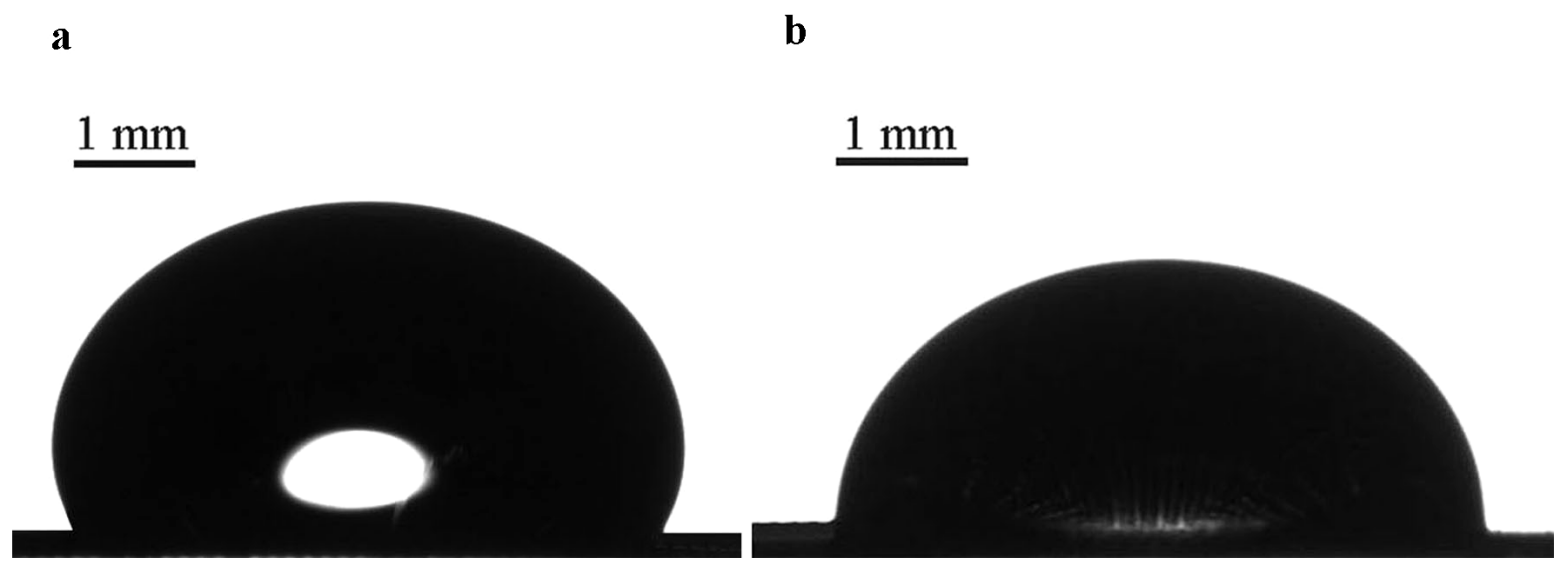
(a) 40 μl droplet of water/ethanol solution deposited on PC-coated gauzes. The obtuse apparent contact angle evidencing a pronounced Cassie-Baxter wetting is clearly seen. (b) The same droplet exposed to the uniform electric field of 2.5 kV/cm. The abrupt change in the apparent contact angle evidencing a wetting transition is seen.

**Figure 7:**
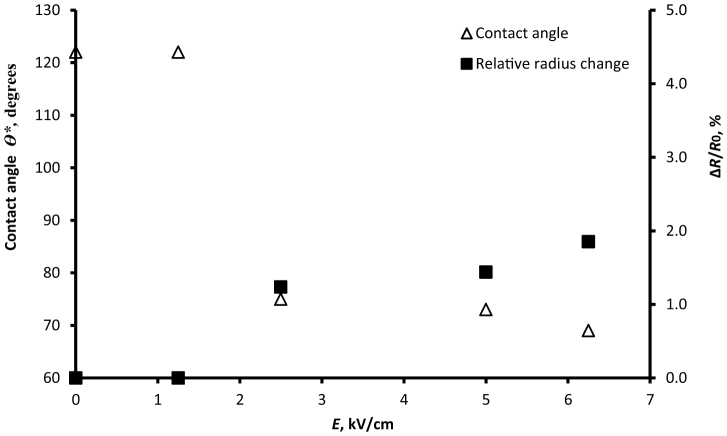
The apparent contact angle θ* and the dimensionless relative radius Δ*R*/*R*_0_ vs. applied electric field *E* for the 20 μl droplet exposed to the uniform electric field.

Membranes were manufactured with the fast dip-coating process. Stainless steel gauzes (depicted in Fig. 2) were used as carrying supports^23^. A 4 wt.% PC solution was prepared by dissolving the polymer in a mixture of chlorinated solvents (chloroform, CHCl_3_, 8 wt.% and dichloromethane CH_2_Cl_2_, 88 wt.%, were supplied by BIO-LAB Ltd, both solvents were of the chemical grade purity). Thoroughly cleaned stainless steel gauzes were pulled vertically at a high speed of *v* = 40 cm·min^−1^ from the polymer solution and dried at room temperature and a rH of 50% in an environmental cell. Rapid evaporation of the solvent cooled the solution/humid air interface subsequently resulting in an intensive condensation of water droplets at the interface^24^,^25^,^26^,^27^,^28^,^29^,^30^. Water droplets then sank into the solution eventually forming micro-scaled pores typical for breath-figures self-assembly^24^,^25^,^26^,^27^,^28^,^29^,^30^. The topography of the membranes was studied with SEM (JEOL JSM 6510 LV, Japan).

Hydrophilization of membranes was achieved by exposure to an inductive cold air plasma discharge under the following parameters: the plasma frequency was on the order of 10 MHz, the power was 20 W, the pressure was *P* = 6.7 · 10^−2^ Pa, the volume of the discharge chamber was 45 cm^3^. The time span of irradiation was 10 s. After treatment, membranes were kept for 150 hours under ambient conditions (24°C and 50% rH) necessary for the hydrophobic recovery occurring after cold plasma treatment^44^,^45^,^46^. The apparent contact angle after completion of the hydrophobic recovery was approximately 90° (mean of 10 measurements).

Distilled water (pH = 6; *σ* = 2.5 μS) was used for the electrowetting-driven water penetration study; a 98 wt.% water/ethanol solution was used for the non-contact scheme. Ethanol (chemical grade, 96% purity) was supplied by BioLab Ltd Israel.

Contact angles were established using a Ramé–Hart goniometer (model 500). Ten measurements were taken to calculate the mean apparent contact angle.

## Author Contributions

E.B. analyzed data and proposed experimental concepts. R.P. planned and carried out experiments. S.B. planned and carried out experiments. D.A. analyzed data. All authors reviewed the manuscript.
